# Korean soybean core collection: Genotypic and phenotypic diversity population structure and genome-wide association study

**DOI:** 10.1371/journal.pone.0224074

**Published:** 2019-10-22

**Authors:** Namhee Jeong, Ki-Seung Kim, Seongmun Jeong, Jae-Yoon Kim, Soo-Kwon Park, Ju Seok Lee, Soon-Chun Jeong, Sung-Taeg Kang, Bo-Keun Ha, Dool-Yi Kim, Namshin Kim, Jung-Kyung Moon, Man Soo Choi

**Affiliations:** 1 National Institute of Crop Science, Rural Development Administration, Wanju-gun, Jeollabuk-do, Republic of Korea; 2 FarmHannong, Ltd., Daejeon, Republic of Korea; 3 Genome Editing Research Center, Korea Research Institute of Bioscience and Biotechnology, Daejeon, Republic of Korea; 4 Department of Bioinformatics, KRIBB School of Bioscience, Korea University of Science and Technology, Daejeon, Republic of Korea; 5 Bio-Evaluation Center, Korea Research Institute of Bioscience and Biotechnology, Cheongju, Chungcheongbuk-do, Republic of Korea; 6 Department of Crop Science & Biotechnology, Dankook University, Cheonan, Chungcheongnam-do, Republic of Korea; 7 Division of Plant Biotechnology, College of Agriculture and Life Sciences, Chonnam National University, Gwangju, Republic of Korea; 8 National Institute of Agricultural Sciences, Rural Development Administration, Jeonju, Jeollabuk-do, Republic of Korea; Central Agricultural University, INDIA

## Abstract

A core collection is a subset that represents genetic diversity of the total collection. Soybean (*Glycine max* (L.) Merr.) is one of major food and feed crops. It is the world’s most cultivated annual herbaceous legume. Constructing a core collection for soybean could play a pivotal role in conserving and utilizing its genetic variability for research and breeding programs. To construct and evaluate a Korean soybean core collection, genotypic and phenotypic data as well as population structure, were analyzed. The Korean soybean core collection consisted of 430 accessions selected from 2,872 collections based on Affymetrix Axiom^®^ 180k SoyaSNP array data. The core collection represented 99% of genotypic diversity of the total collection. Analysis of population structure clustered the core collection into five subpopulations. Accessions from South Korea and North Korea were distributed across five subpopulations. Analysis of molecular variance indicated that only 2.01% of genetic variation could be explained by geographic origins while 16.18% of genetic variation was accounted for by subpopulations. Genome-wide association study (GWAS) for days to flowering, flower color, pubescent color, and growth habit confirmed that the core collection had the same genetic diversity for tested traits as the total collection. The Korean soybean core collection was constructed based on genotypic information of the 180k SNP data. Size and phenotypic diversity of the core collection accounted for approximately 14.9% and 18.1% of the total collection, respectively. GWAS of core and total collections successfully confirmed loci associated with tested traits. Consequently, the present study showed that the Korean soybean core collection could provide fundamental and practical material and information for both soybean genetic research and breeding programs.

## Introduction

Genetic diversity is fundamental in crop breeding and research programs. Although genetic diversity could be a barrier to progress, it is essential for crop improvement. Monoculture of a few improved cultivars has decreased the genetic diversity across crop species [1]. One of important ways to achieve successful crop improvement is through continuous supply of genetic diversity, including new or improved variability in target traits [2]. Therefore, managing and utilizing large and diverse germplasm collection are important and valuable challenges for successful biological research and crop improvement programs.

Across the world, germplasm conservation centers have been established to preserve genetic diversity of target crop species. Although large numbers of collections are desirable to preserve genetic variability, their usefulness and accessibility might be inversely related to their size [3]. An increase in the number of accessions without detailed information could decrease the efficiency and value of these collections [4, 5]. To overcome the size issue, core collections have been constructed for important crop species. A core collection was first defined as “a limited set of accessions” representing genetic diversity with minimum repetitiveness in the total collection [1, 6, 7]. Presently, core collections for approximately 30 species have been constructed, including rice (*Oryza sativa* L.) [8], wheat (*Triticum aestivum* L.) [9], cotton (*Gossypiumbar badense* L.) [10], peanut (*Arachis hypogea* L.) [11], pepper (*Capsicum* spp.) [12], jujube (*Ziziphus jujube* Mill.) [13], melon (*Cucumis melo* L.) [14], medicago (*Medicago* spp.) [15], and soybean (*Glycine max* L. Merr.) [16].

Soybean is one of the most important crop species as both food and feed sources in the world. Soybean genetic resources are largely preserved in China, the USA, Japan, and Korea [6, 17, 18, 19, 20]. In Korea, approximately 20,000 accessions of soybean are maintained in the National Agrobiodiversity Center of Rural Development Administration (http://genebank.rda.go.kr/). China has the largest soybean collection. There are approximately 26,000 accessions in the Institute of Crop Germplasm Resources of the Chinese Academy of Agricultural Science [19, 20]. The United States Department of Agriculture (USDA) has nearly 17,000 soybean accessions [6]. In Japan, around 11,000 soybean accessions are maintained at the National Institute of Agrobiological Sciences (NIAS) Genebank [17].

Many studies have evaluated various strategies to construct core collections of many crop species [6, 17, 18, 21, 22]. These strategies include random, proportion, constant, logarithmic, and genetic diversity-based methods. Among them, genetic diversity-based method has been considered the simplest and the most efficient [21]. Through genetic diversity-based strategy using 48 SNPs, Lee et al. [12] have elected 240 accessions from 3,821 capsicum collections. Xu et al. [13] have selected a core collection consisting of 150 accessions from 947 jujube collections using 24 SSR markers. However, considering genome sizes of crop species, the above studies used relatively few genetic markers to select accessions for core collections.

Although a core collection of soybean was first constructed in 1987 [23], no follow-up study using this collection has been reported. A Chinese soybean core collection has been established from 23,857 collections based on agronomic traits and SSR markers [16]. Cho et al. [24] have reported a core collection of Korean landraces selected from around 7,000 collections. Oliveira et al. [6] have developed a soybean core collection of the USDA Soybean Germplasm Collection by using several sampling strategies. A Japanese mini-core collection consisting of 96 accessions has been selected by using 191 SNP markers from 1,603 collections [17]. Priolli et al. [25] have developed a core collection with 31 accessions from 435 Brazilian soybean cultivars using 27 SSR markers. Kuroda et al. [22] have developed two core collections from 1,359 wild and cultivated soybean collections using information of 20 SSR markers. Likewise, genetic diversity analyses have been performed to construct core collections in soybean by using phenotypic information or a small number of SSR markers. In recent years, genetic markers have been increasingly developed and applied to many crop species, including barley [26], wheat [27], common bean [28], maize [29], sunflower [30], apple [31], tomato [32], and soybean [33].

The objectives of this study were to: 1) construct a Korean soybean core collection from 2,872 soybean collections, 2) evaluate genetic and phenotypic diversity of core and total collections, and 3) conduct genome-wide association studies (GWAS) for several important phenotypic traits in both core and total collections and compare GWAS results.

## Material and methods

### Plant materials

A total of 2,872 soybean collections (total collection) maintained in the National Agrobiodiversity Center in the Rural Development Administration (Jeonju, S. Korea) were used for this study. These soybean collections originated from China, Japan, the USA, N. Korea, S. Korea, and other countries (Canada, France, Sweden, and unknown). Among these collections, 2,872 cultivated soybeans originated from China, Japan, the USA, N. Korea, S. Korea, and others (76, 41, 79, 108, 2,556, and 12 accessions, respectively).

### DNA extraction and SNP genotype analysis

Young trifoliate leaves from a single plant of each collection were collected for DNA extraction. Genomic DNA was isolated using the CTAB (cetyltrimethylammonium bromide) method [34] with a minor modification. Axiom^®^ 180k (180,961) SoyaSNP array was used for SNP genotyping [35]. Genomic DNAs from the total collection were hybridized to arrays using Affymetrix GeneTitan system based on the manufacturer’s instructions. SNP genotyping was conducted based on Axiom^®^ Genotyping Solution Data Analysis User Guide [35].

### Genetic diversity analysis of the total collection

Phylogenetic analysis of the total collection was performed using genotype data from the Axiom^®^ 180k SoyaSNP array. Phylogenetic tree and principal component analysis (PCA) were conducted by using APE R package [36]. A distance matrix was calculated from pairwise DNA sequences of samples. Phylogenetic tree was constructed based on the distance matrix using a neighbor-joining algorithm. The distance matrix was also used for PCA. To estimate population structure of a core collection, fastSTRUCTURE [37] was used. This program has been widely used to calculate posterior inference based on the Bayesian framework [38, 39]. SNP genotype data set from 2,872 soybean accessions were converted to variant call format (VCF) format and fastSTRUCTURE was executed for various numbers of populations (K = 1,…,10). Numbers of subpopulations were defined using the marginal likelihood function. Arlequin v 3.5 [40] program was used to compare molecular diversity between and within subpopulations and geographic regional groups using analysis of molecular variance (AMOVA) [41, 42].

### Construction of a core collection

To select accessions for a core collection, genotype data of the total collection were analyzed using GenoCore [43], a fast and consistent method for large datasets. GenoCore offers two options: coverage and delta. The coverage option provides a percentage value representing how many of the selected subsets of samples reflect the diversity of the total collection while the delta value represents increasing ratio in the coverage. In the present study, 99% of the coverage and 0.001% of the delta value were applied.

### Evaluation of phenotypic traits

Five qualitative traits and two quantitative traits were evaluated for both core and total collections in field tests. The total collection was tested in an experimental field at the National Institute of Crop Science (Suwon, S. Korea, 37°15’29.1” N, 126°58’34.3” E) in 2013 and the core collection was grown in an experimental field at the National Institute of Crop Science (Wanju, S. Korea, 35°50’35.2” N, 127°02’45.9” E) in 2016. The experimental design was a randomized block design (RBD) with two replicates. The field plot was 3 m long with 0.75 m of row spacing and plant spacing within rows of 0.15 m. Two seeds were sown per spot (or hole).

Five qualitative traits, including hypocotyl color, leaflet shape, flower color, growth habit, and pubescent color, were investigated at V1, R1, R2, R3, and R7 stages, respectively. Flower color was recorded as purple or white. Hypocotyl color was purple or green. Pubescent color was tan or gray. Growth habit was recorded as either determinate or indeterminate type. Leaflet shape was classified as ovate or narrow. In the case of quantitative traits, days to flowering (R1) were recorded as numbers of days from planting to beginning to bloom. Each accession was individually bulk-harvested after its full maturity stage (R8). A sample of 100 cleaned seeds from each collection was randomly selected and weighed.

### Genome-wide association study

To evaluate whether the core collection could sufficiently represent genetic diversity of the total collection, four phenotypic traits (days to flowering, flower color, pubescent color, and growth habit) with known controlling genes were tested through GWAS. After filtration of SNPs with < 1% minor allele frequency (MAF) and > 5% heterozygosity, 131,620 SNPs were selected and used for GWAS using a compressed mixed linear model [44]. All analyses were conducted using GAPIT [45] and GenABEL package [46] in R Project (http://www.r-project.org/). GWAS was conducted for both core and total collections to compare their genetic diversities for tested phenotypic traits.

Genes controlling flower color (W1), pubescent color (T), and growth habit (TFL1) of soybean have been mapped to chromosome 13, 6, and 19, respectively [47, 48, 49]. Quantitative trait loci (QTL) associated with days to flowering, named *E1*, *E2*, and *E3*, have been genetically mapped to chromosome 6, 10, and 19 in soybean, respectively [50, 51, 52, 53]. Above-listed traits were analyzed for both core and total collections.

## Results

### Soybean germplasm genotyping

To identify genetic diversity in the total collection, 170,223 high-quality SNPs from the Axiom^®^ 180k SoyaSNP array were used. In the total collection, average sample call rate, homozygosity rate, and heterozygosity rate were 99.41%, 98.82%, and 0.59%, respectively. In the core collection, average sample call rate, homozygosity rate, and heterozygosity rate were 98.83%, 97.21%, and 1.62%, respectively (Table 1). Soybean accessions with call rates less than 97% were excluded from future analysis (Affymetrix, Analysis Guide Axiom^®^ Genotyping solution Data Analysis Guide).

**Table 1:** Genotypic diversity of 2,872 accessions in the total collection and 430 accessions in the core collection by Axiom^®^ 180K SoyaSNP array.

	Sample call rate (%)			Homozygosity rate (%)			Heterozygosity rate (%)			Ratio of sample call rate higher than 97% (%)
	Max.	Min.	Aver.	Max.	Min.	Aver.	Max.	Min.	Aver.	
**Total** **collection**	99.95	49.31	99.41	99.82	26.63	98.82	23.00	0.12	0.59	98.00
**Core** **collection**	99.95	55.13	98.83	99.82	33.24	97.21	21.89	0.13	1.62	98.84

### Construction of the core collection

The 170,223 high-quality SNPs were also used to construct the core collection from the total collection (S1 Table). Based on genotyping data, accessions with more than 99.9% similarity or with SNPs ≤ 1% of MAF were excluded from further analysis.

We first selected 407 accessions reflecting 99% of genetic diversity of total accessions. Second, as additional sources of genetic variations, 24 cultivated soybeans used for whole-genome re-sequencing were included with the first selection. This addition produced a core collection consisting of 431 soybean accessions. However, we found that one collection (Danbaek) was included in both the first selection and the re-sequencing accessions. Finally, a Korean soybean core collection consisting of 430 accessions was constructed (Table 2 and S2 Table).

**Table 2:** Construction of the core collection by using Axiom^®^ 180K SoyaSNP array and GenoCore.

Origin	Total collection		Core collection		
	Before redundant germplasm removal	After redundant germplasm removal	Extraction by GenoCore	Added re-sequencing resources	Final core collection
**South Korea**	2,556	2,236	298	22 (-1)*	319
**North Korea**	108	68	41	0	41
**China**	76	36	41	1	42
**Japan**	41	27	13	0	13
**USA**	79	66	11	1	12
**The other countries**	12	9	3	0	3
**Total**	**2,872**	**2,442**	**407**	**24**	**430**

*(-1): one duplicate resource among added whole-genome re-sequencing resources was excluded.

**Fig 1:**
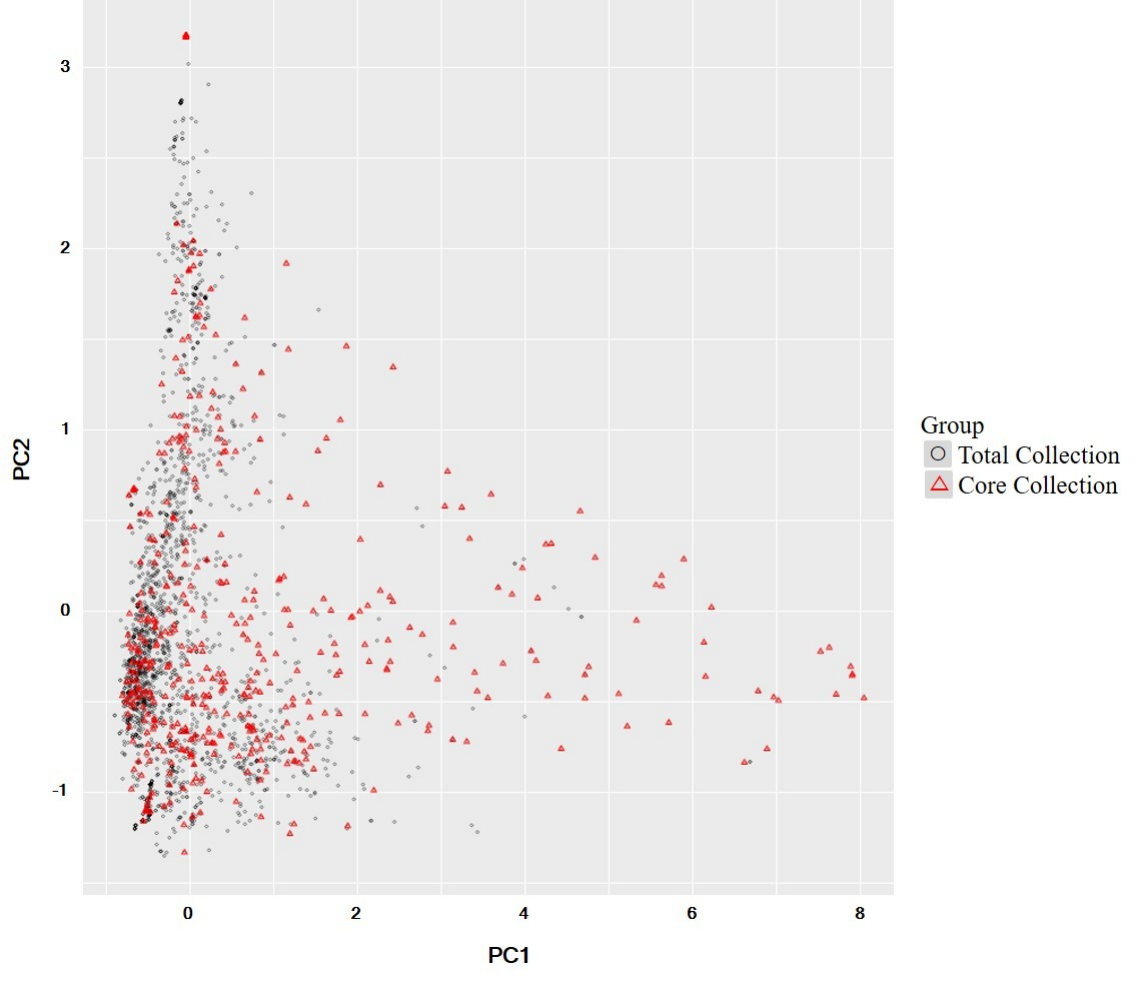
Principal component analysis of the core collection (red triangle) and the total collection (black circle).

**Fig 2:**
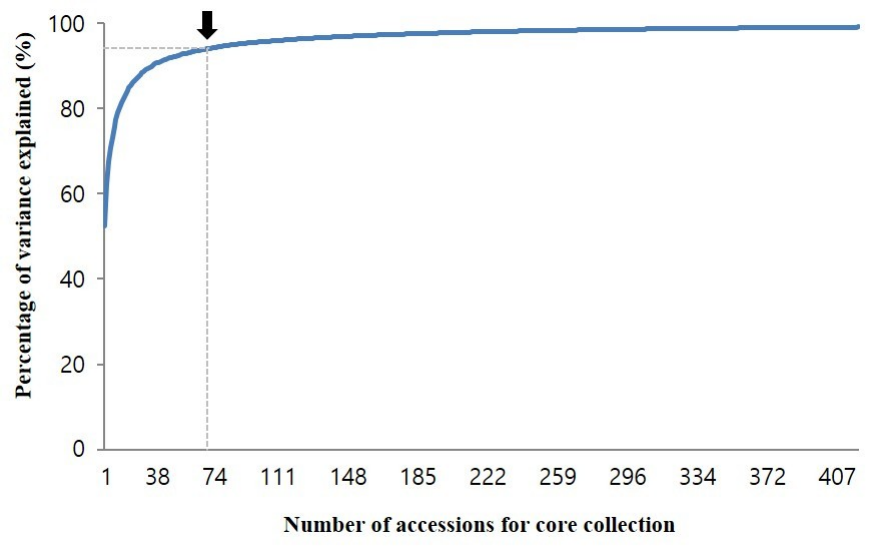
Principal component analysis model statistics for the core collection. The core collection was made of 407 accessions, reflecting more than 99% of the genetic diversity of the total collection. Specifically, 72 accessions reflected more than 95% of the genetic variations of the total collection (black arrow).

PCA was conducted to confirm whether the core collection represented the genetic diversity of the total soybean collection. Results showed that the distribution of collections by genetic diversity showed similar trends for both core and total accessions (Fig 1). As shown in the scree plot, genetic variations of 72 accessions reflected more than 95% of genetic variations in the total collection (Fig 2). In addition, 407 accessions reflected more than 99% of genetic variations of the total collection (S2 Table). The number of accessions in the core collection accounted for about 15% of the total collection. Accessions in the core collection originated from South Korea (319, 74%), North Korea (41, 9%), China (42, 10%), Japan (13, 3%), the USA (12, 3%), and other countries (3, 1%) (Table 2).

### Population structure and phylogenetic analysis

**Fig 3:**
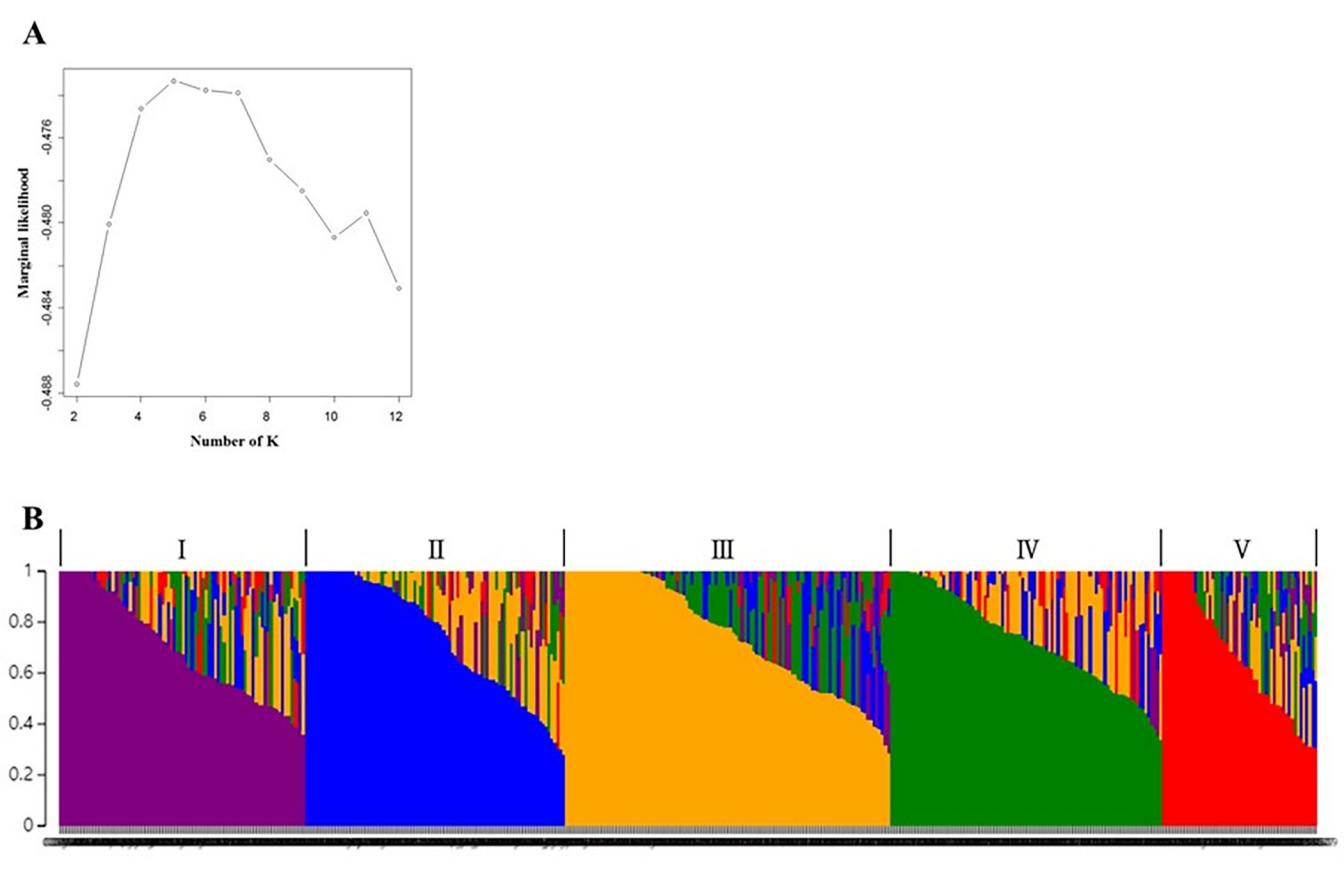
Population structure of the core collection. (A) The number of populations (K = 5) was selected using marginal likelihood provided by fastSTRUCTURE. (B) Five subpopulation clusters inferred by fastSTRUCTURE are indicated by different colors.

SNP information was used to evaluate the population structure of the core collection. Marginal likelihood was found to be the highest when K was 5 (Fig 3A). The number of accessions belonging to each subpopulation was as follows: subpopulation I, 84; subpopulation II, 89; subpopulation III, 111; subpopulation IV, 93; and subpopulation V, 53. AMOVA indicated that only 2.01% of the molecular variance was explained by the origin while 16.18% was contributed by the subpopulation. The variation among individuals within populations was the highest (81.82%, Table 3). Accessions from South Korea and North Korea were distributed across five subpopulations while 30 of 42 collections from China belonged to subpopulation V. Most of the improved lines belonged to subpopulations I and II while most of landraces belonged to subpopulations III and IV.

**Table 3:** Analysis of molecular variance of geographic groups and five subpopulations in the Korean core collection.

Source of variation	d.f.^a^	Sum of squares	Variance components	Percentage of variation	Fixation indices^b^	*P*-value
Among groups	4	425006.999	286.71352	2.01	*F*_*CT*_ = 0.02006	0.10655
Among populations within groups	16	1628562.092	2312.39583	16.18	*F*_*SC*_ = 0.16509	< 0.0001
Within populations	839	9811777.546	11694.60971	81.82	*F*_*ST*_ = 0.18186	< 0.0001
Total	859	11865346.637	14293.71907			

^a^d.f.: degrees of freedom.

^b^Fixation indices: *F*_*CT*_, difference among groups, *F*_*SC*_, difference among populations within groups; *F*_*ST*_, difference among populations.

**Fig 4:**
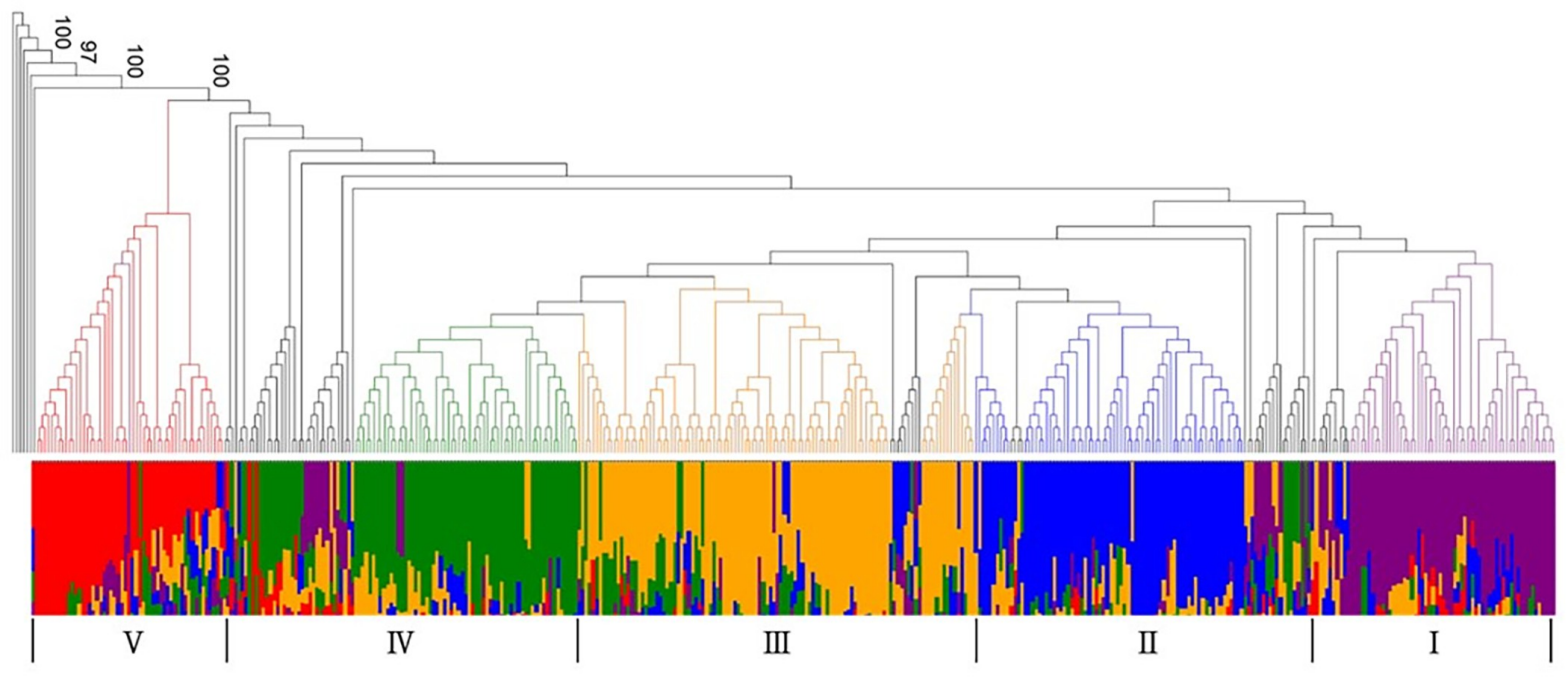
Phylogenetic tree of the core collection. The core collection was divided into five subpopulation clades (I-V).

**Fig 5:**
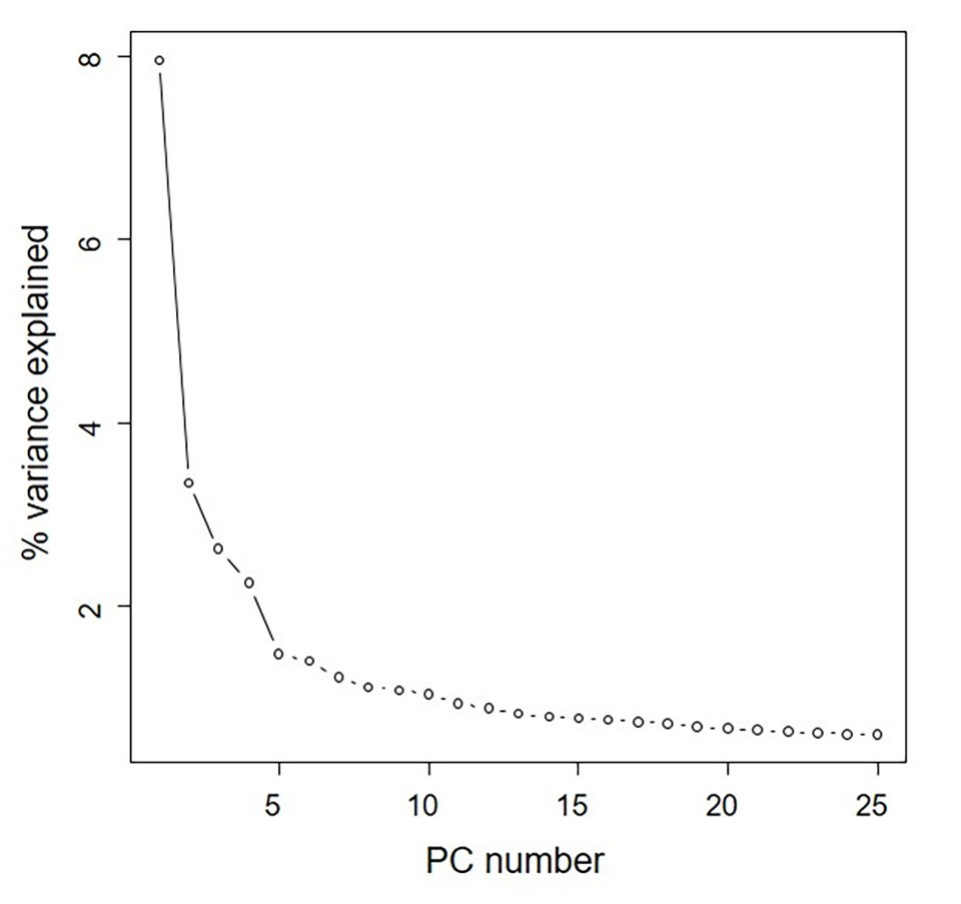
Scree plot of principal component analysis in the core collection between variance and number of principal components.

**Fig 6:**
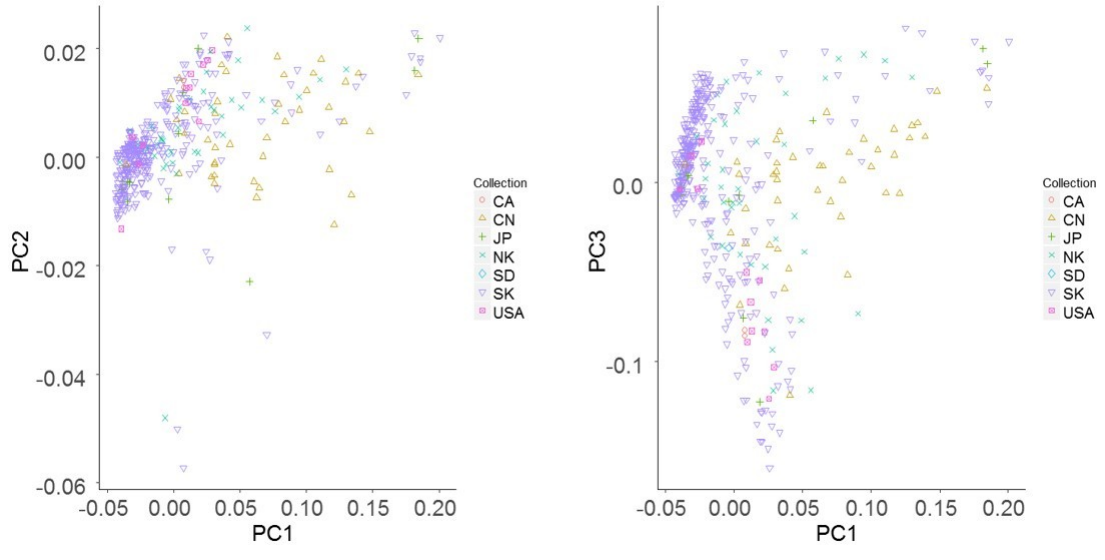
Principal component analysis of the Korean core collection. CA: Canada; CN: China; JP: Japan; NK: North Korea; SD: Sweden; SK: South Korea; USA: United States of America.

The population structure of the core collection was doubled-checked using both phylogenetic tree and PCA. In the phylogenetic tree, most accessions with more than 50% admixture fractions for a genome were distributed over the five subpopulations while admixed accessions were placed between subpopulation clades (Fig 4). Scree plot showed that five subpopulations were optimal (Fig 5). PCA plots indicated that accessions from the South and North Korea were evenly distributed and 28 out of 42 Chinese accessions (67%) were grouped together (Fig 6). As a result, although most of the resources in the Korean soybean core collection were Korean soybean resources and evenly distributed, the variation of Korean resources did not cover variation in Chinese resources.

### Phenotypic variations in the core collection

**Fig 7:**
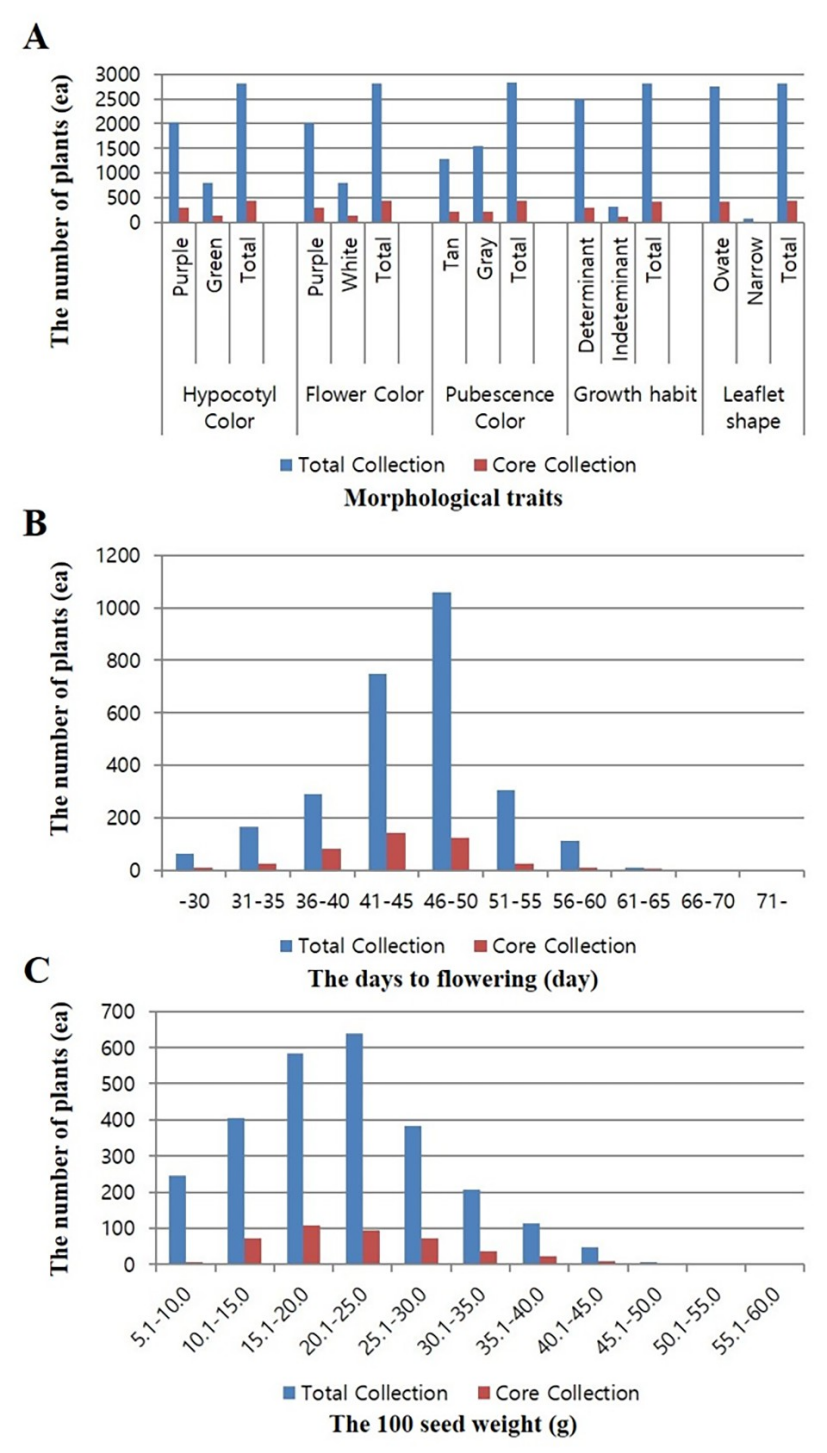
Morphological trait measurements for the core collection and the total collection. (A) Flower color, hypocotyl color, pubescent color, growth habit, and leaflet shape in the core collection and total collection. (B) Days to flowering in the core collection and total collection. (C) The 100-seed weight in the core collection and the total collection.

Important phenotypic traits of soybean in both core and total collections were evaluated. In the core collection, days to flowering ranged from 27 to 68 days (Fig 7B). Two hundred ninety-seven accessions had purple color hypocotyls and flowers while 133 had green hypocotyls and white flowers. For pubescent color, 210 accessions were tan and 220 were gray. Regarding growth habit, 305 accessions presented a determinate growth type while 121 accessions had an indeterminate type. Growth habits of four accessions were not noted. Most accessions (412) had ovate leaflet shapes while only 18 had narrow leaflet shapes (Fig 7A and S3 Table). The 100-seed weight of these accessions ranged from 7.3 to 59.4 g (Fig 7C).

In the present study, we investigated and compared five phenotypes in core and total collections. The proportion of each trait (flower color, hypocotyl color, pubescent color, growth habit, and leaflet shape) in the core collection to that in the total collection was approximately 18.1%. Specifically, proportions of these qualitative traits in the core collection to that in the total collection were around 15.8% in South Korea, 38% in North Korea, 51.4% in China, 27.7% in Japan, 16.6% in the USA, and 20.2% in other countries (Fig 7A and S3 Table). These results indicated that phenotypic proportions of the tested phenotypic traits in the core collection were similar to those in the total collection, although the core collection was selected based on SNP genotypes.

In the case of quantitative traits, the proportion of days to flowering in the core collection to that in the total collection was around 26.1%. The proportion of days to flowering in the core collection to that in the total collection was around 12.1% in S. Korea, 48.9% in N. Korea, 68.6% in China, 47.8% in Japan, 19.7% in the USA, and 39.3% in other countries (Fig 7B and S4 Table). The proportion of the 100-seed weight in the core collection to that in the total collection was about 25.2%, 23.7% in S. Korea, 33.3% in N. Korea, 77.9% in China, 37.5% in Japan, 24.1% in the USA, and 100% in other countries (Fig 7C and S5 Table). The 100-seed weight in the total collection ranged from 6.2 g to 59.4g. Similarly, the 100-seed weight in the core collection ranged from 7.3 g to 59.4g. Days to flowering ranged from 21 to 78 days in the total collection and from 27 to 68 days in the core collection.

We also compared the distribution of accessions between total and core collections by their origins (S1, S2, and S3 Figs). The distribution trend of the accessions by origin in the core collection was similar to that in the total collection. In particular, the percentage of the core collection extracted from the total collection of Chinese resources was the highest (qualitative traits, 51.4%; days to flowering trait, 68.6%; 100-seed weight trait, 77.9% for Chinese resources extracted from the total collection) (S1C, S2C, and S3C Figs). These results might indicate that the genetic diversity of Chinese collections was higher than that of collections from other countries.

### Genome-wide association study

**Fig 8:**
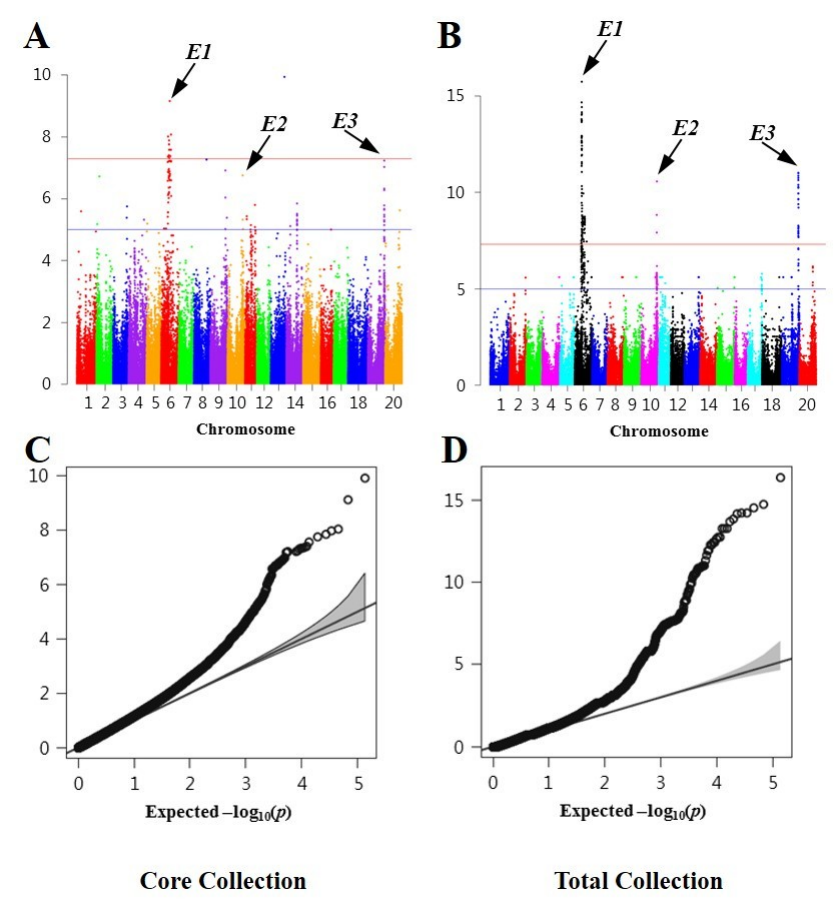
GWAS for days to flowering in the core collection and the total collection. (A) Manhattan plot of the core collection. (B) Manhattan plot of the total collection. (C) Quantile-Quantile plot for days to flowering in the core collection. (D) Quantile-Quantile plot for days to flowering in the total collection.

Results of GWAS for the four important phenotypic traits in core and total collections were compared. For flower color (W1), pubescent color (T), and growth habit (TFL1), GWAS confirmed that both collections had significant SNPs at gene regions of the same positions. QTLs associated with days to flowering genes *E1*, *E2*, and *E3* were positioned on the same chromosomes in both core and total collections (Fig 8).

## Discussion

In the present study, a Korean soybean core collection was constructed based on SNP information from the Axiom^®^ 180k SoyaSNP array. Genotypic, phenotypic diversity, and population structure of the population were then analyzed. As mentioned previously, earlier studies have tested various selection methods to construct core collections in diverse crop species. To date, most of core collections for crop species have been developed based on geographic origin, morphological, and phenotypic traits. Ultimate goals of a core collection are not only to have the maximum possible genetic diversity of the total collection, but also to have minimum repetition. However, it has been reported that phenotypic data cannot perfectly reflect the genetic diversity of the total collection since most of quantitative agronomic traits are easily affected by environmental factors [54]. In contrast, molecular markers can directly reflect the genetic diversity in a total collection at DNA sequence level [55].

Although several studies have previously established core collections based on genotypic data, relatively few molecular markers have been used to determine genetic diversity in total collections and select core collections. Kuroda et al. [22] have used 20 SSR markers to develop two core collections of wild and cultivated soybean. To develop a core set from 435 Brazilian soybean cultivars, Priolli et al. [25] have used 130 alleles from 27 SSR loci. Xu et al. [13] have used 24 SSR markers to construct the Chinese jujube (*Ziziphus jujube* Mill) core collection. For *Capsicum* germplasm, Lee et al. [12] have constructed a core collection ‘CC240’ consisting of 240 accessions by using 48 SNP markers and 32 phenotypic/morphological traits. The number of SNP markers (170,223) used to construct the core collection in the present study was significantly greater than that used in previously reported studies. It sufficiently covered all genomic regions of soybean. In addition to high-density molecular markers, the use of precisely tested phenotypic and agronomic traits can improve the quality of selection strategies used to construct core collections in crop species. After the development of a core collection, accurate evaluation of important and target phenotypes of the core collection in multiple environments will accelerate the course of gene discovery and marker development.

Compared to STRUCTURE analysis, the phylogenetic tree did not present clear separation according to geographical origin. In particular, most of the Chinese collections belonged to subpopulation V (S2 Table). However, collections from other countries were evenly distributed across subpopulations in both the phylogenetic tree and the population structure analysis. AMOVA indicated that genetic variability of populations within groups was higher than that between groups. Although few accessions from other countries were included in the core collection and the number of accessions from Korea was larger than that from other countries, the genetic diversity of Korean accessions was significantly higher than that of accessions from other countries. In other words, there is genetic distance between resources from Korean and other countries. If a new core collection was constructed by integrating the core collection developed from China, Japan, USA, and Korea, genetic distances between the collections from each origin would be clearly elucidated.

The ultimate aim of constructing the Korean soybean core collection in this study was to improve the utilization of useful breeding materials for genome prediction or selection in soybean breeding programs as well as to explore candidate genes related to particular traits through GWAS. In the present study, GWAS was performed to confirm whether the core collection represented genetic diversity of the total collection. Monogenic traits including flower color (*W1*), pubescent color (*T*), and growth habit (*TFL1*) were identically detected in both collections. However, the GWAS for polygenic traits presented different results for the two collections. In the case of *E1* (major gene for days to flowering), GWAS results were identical for the two collections. The *p*-value of the SNP associated with *E1* was highly significant in the two collections: *p* = 1.83E-08 in the core collection and *p* = 1.21E-11 in the total collection. In the case of minor genes (*E2* and *E3*), GWAS results for *E3* were similar for the two collections. The *p*-value of the SNP representing *E3* was also highly significant in the two populations: *p* = 3.67E-07 in the core collection and *p* = 5.40E-10 in the total collection. However, the *p*-value of the SNP representing *E2* was found to be highly significant (*p* = 3.81E-12) in the total collection, but less significant (*p* = 0.0021) in the core collection (S6 Table). These results indicated that the size of the populations might be an important factor in GWAS for minor QTLs and that the Korean core collection was suitable for GWAS.

Approximately 10% of the total collection has been recommended for the size of a core collection [1]. Most of the core collections surveyed by Spillane et al. (unpublished) were also 5–20% of the size of the total collection [56]. A small-sized population might not identify QTLs with minor effects. However, the size of the core collections could be determined by genetic diversity of the total population. If the genetic diversity of the total population is low, the size of the core collection does not need to be large. In contrast, the size of the extracted core collection should be large if the genetic diversity of the total population is high. Therefore, we strongly recommend extracting core collections from large accessions with extensive genetic diversity. After developing core collections for crop species, utilization of the collections will become more and more important.

In conclusion, in the work, we constructed a Korean soybean core collection representing genetic and phenotypic diversity of the total collection based on the 180K SNP genotypes. Several genetic studies and breeding programs using core collection are currently ongoing to identify tolerances to biotic and/or abiotic stresses, find candidate genes for important agricultural traits, develop molecular markers for these traits, and develop marker sets for genome selection. The Korean soybean core collection can provide useful genetic material for soybean diversity and genetic studies as well as effective information for soybean breeding programs. Detailed information on soybean accessions included in this Korean core collection is available from the corresponding authors. It can also be obtained from the National Agrobiodiversity Center, Rural Development Administration through its website at http://genebank.rda.go.kr.

## Supporting information

S1 Fig Comparison of the five morphological traits in the core collection and the total collection by origin. (TIF)

S2 Fig Comparison of days to flowering between the core collection and the total collection by origin. (TIF)

S3 Fig Comparison of the 100-seed weight between the core collection and the total collection by origin. (TIF)

S1 Table List of the total collection. (XLSX)

S2 Table List of the core collection. (XLSX)

S3 Table Morphological traits investigated in the core collection and total collection. Tested traits included flower color, hypocotyl color, pubescent color, growth habit, and leaflet shape.
(XLSX)

S4 Table Investigation of days to flowering in the core collection and the total collection. (XLSX)

S5 Table Investigation of 100-seed weight in the core collection and the total collection. (XLSX)

S6 Table Information on SNPs linked to days to flowering (*E1*, *E2*, and *E3*) from GWAS. (XLSX)
