# Supplementary figures and images for: Korean soybean core collection: Genotypic and phenotypic diversity population structure and genome-wide association study
Source: PLoS One. 2019 Oct 22;14(10):e0224074. doi: 10.1371/journal.pone.0224074 (PMC6804985; doi:10.1371/journal.pone.0224074)

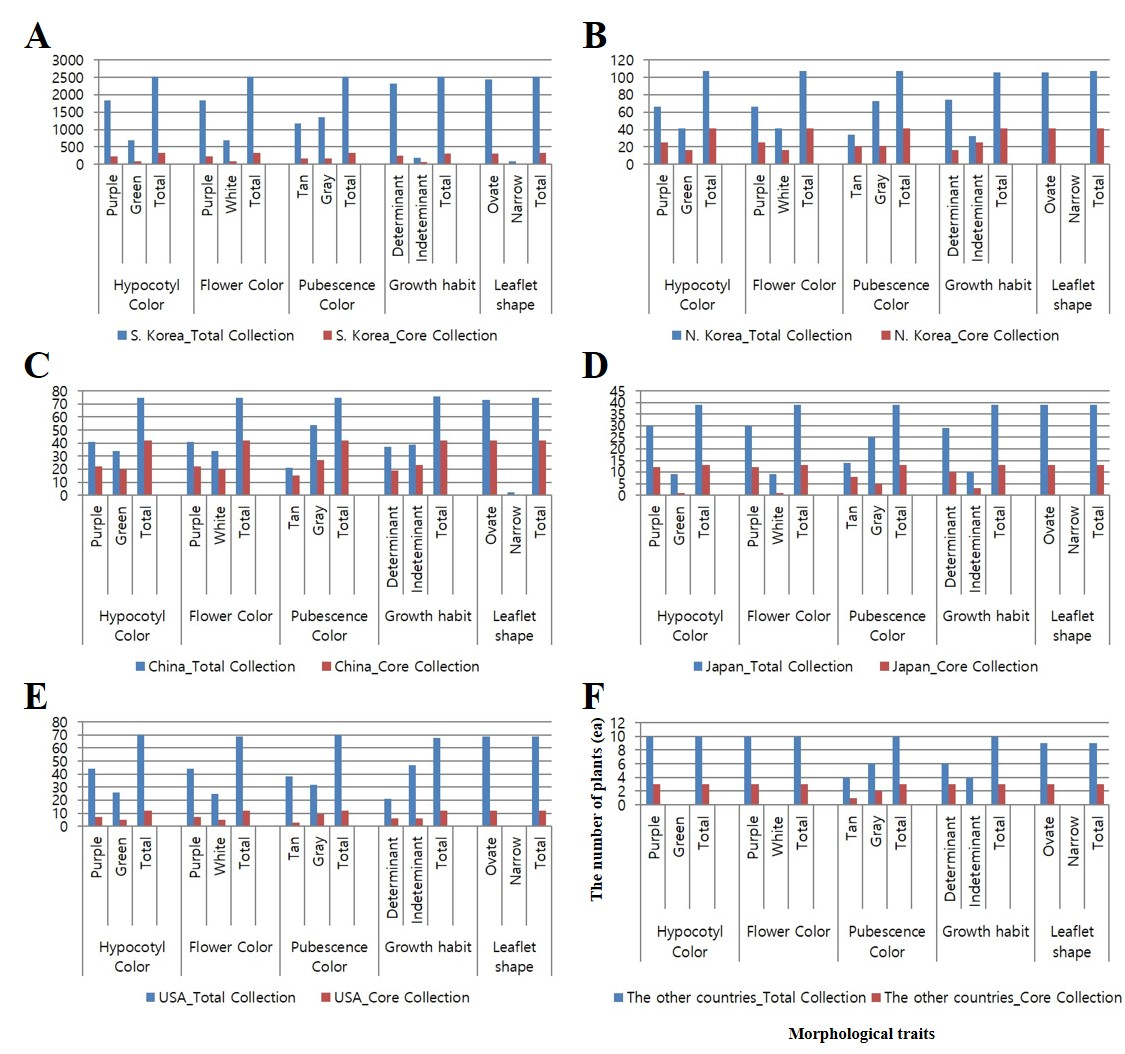

Supplement: S1 Fig — (TIF) [file pone.0224074.s001.tif]

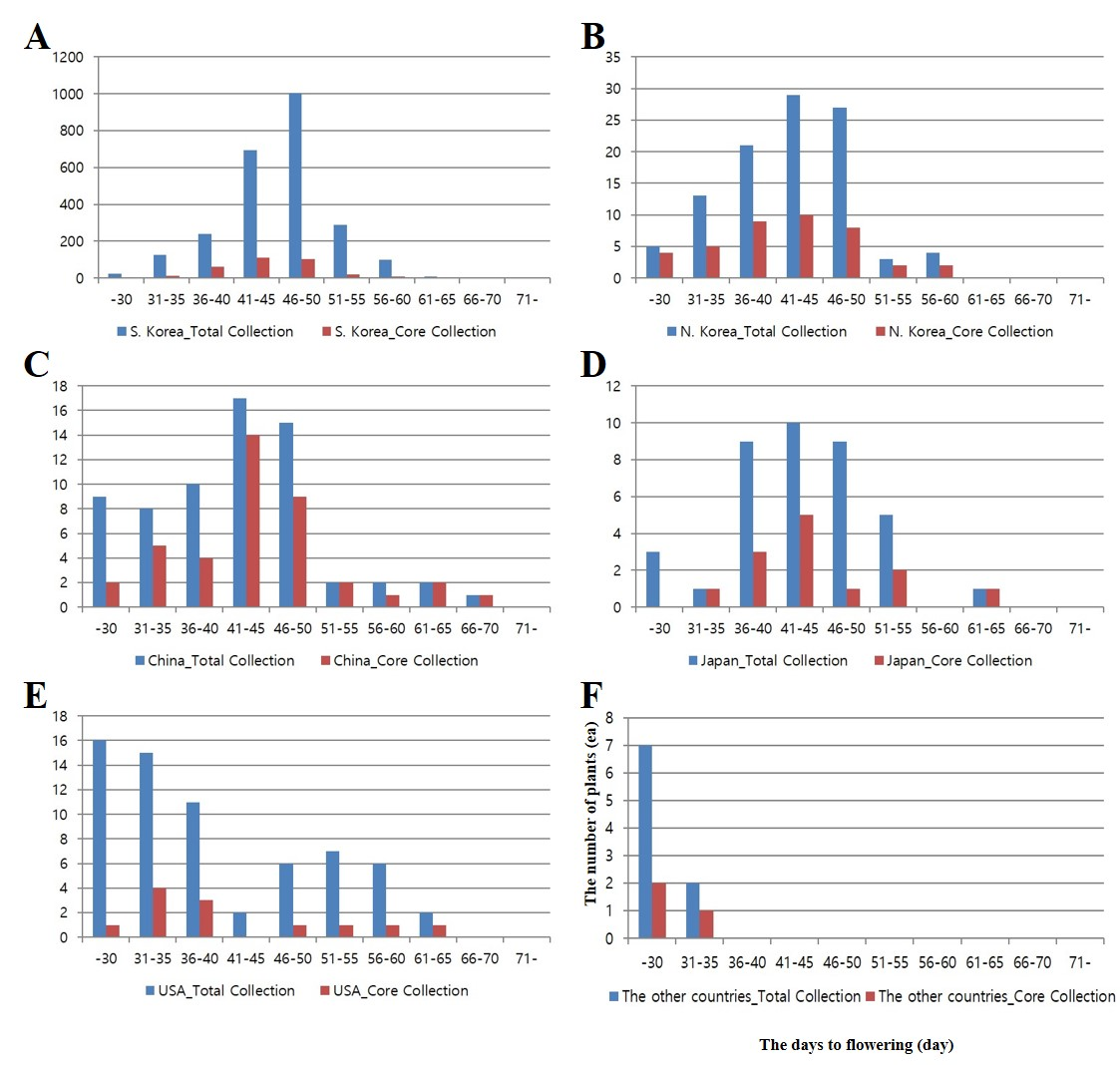

Supplement: S2 Fig — (TIF) [file pone.0224074.s002.tif]

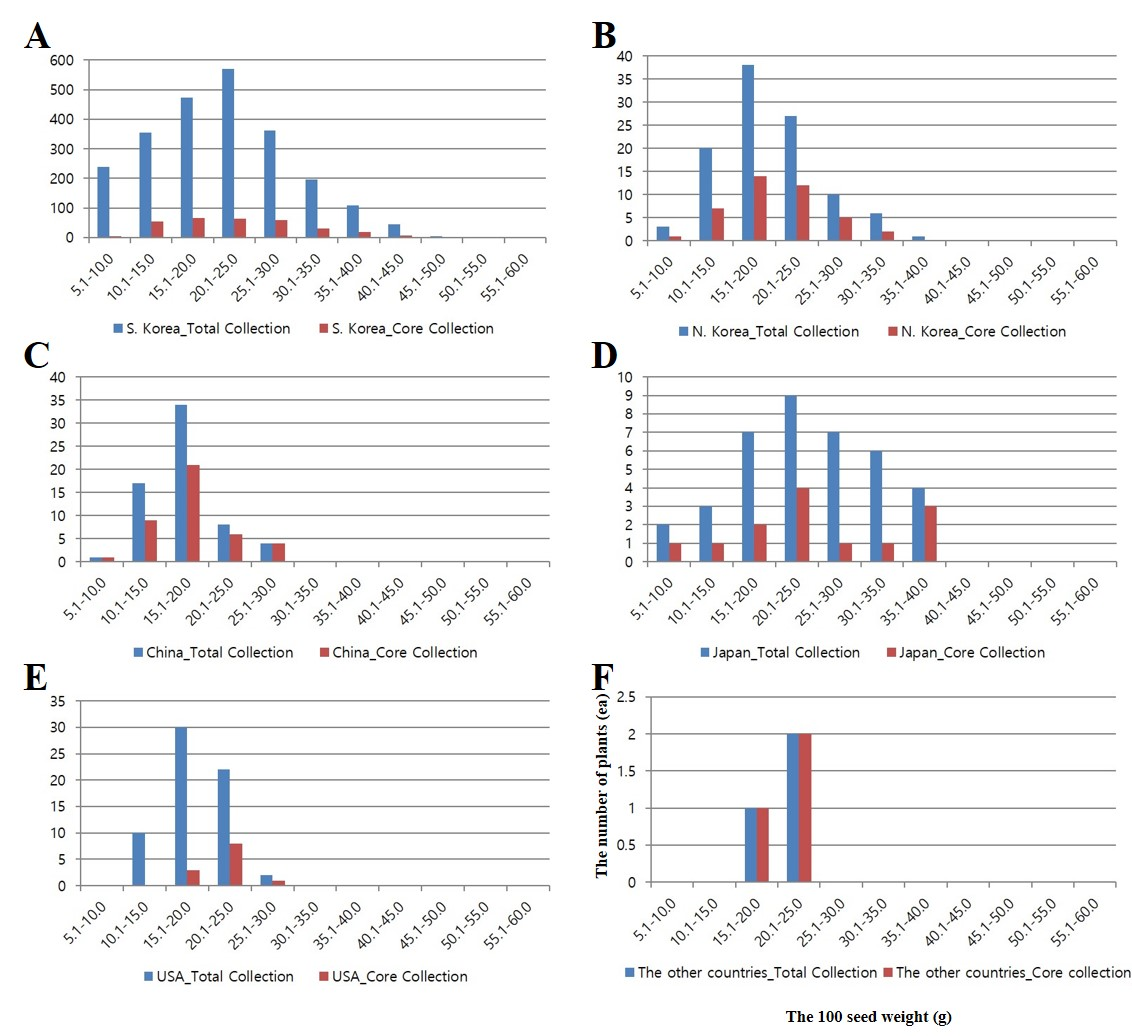

Supplement: S3 Fig — (TIF) [file pone.0224074.s003.tif]
